# Identification of an unfolded protein response-related signature for predicting the prognosis of pancreatic ductal adenocarcinoma

**DOI:** 10.3389/fonc.2022.1060508

**Published:** 2023-01-13

**Authors:** Lishan Fang, Shaojing Chen, Hui Gong, Shaohua Xia, Sainan Guan, Nali Quan, Yajie Li, Chao Zeng, Ya Chen, Jianhang Du, Shuguang Liu

**Affiliations:** ^1^ Department of Medical Research Center, The Eighth Affiliated Hospital, Sun Yat-Sun University, Shenzhen, China; ^2^ Department of Laboratory Medicine Center, Huazhong University of Science and Technology Union Shenzhen Hospital and the 6th Affliated Hospital of Shenzhen University, Shenzhen, China; ^3^ Department of Gastrointestinal Endoscopy Center, The Eighth Affiliated Hospital, Sun Yat-Sun University, Shenzhen, China; ^4^ Department of Ultrasound Imaging, The Eighth Affiliated Hospital, Sun Yat-Sun University, Shenzhen, China; ^5^ Department of Clinical Laboratory, The Eighth Affiliated Hospital, Sun Yat-Sun University, Shenzhen, China; ^6^ Department of Orthopedics, Shenzhen Third People’s Hospital and the Second Affiliated Hospital of Southern University of Science and Technology, Shenzhen, China; ^7^ Department of Pathology, The Eighth Affiliated Hospital, Sun Yat-Sun University, Shenzhen, China

**Keywords:** unfolded protein response, risk score, pancreatic cancer, prognostic model, survival analysis

## Abstract

**Background:**

Pancreatic ductal adenocarcinoma (PDAC) is a highly aggressive lethal malignancy. An effective prognosis prediction model is urgently needed for treatment optimization.

**Methods:**

The differentially expressed unfolded protein response (UPR)‒related genes between pancreatic tumor and normal tissue were analyzed using the TCGA-PDAC dataset, and these genes that overlapped with UPR‒related prognostic genes from the E-MTAB-6134 dataset were further analyzed. Univariate, LASSO and multivariate Cox regression analyses were applied to establish a prognostic gene signature, which was evaluated by Kaplan‒Meier curve and receiver operating characteristic (ROC) analyses. E‒MTAB‒6134 was set as the training dataset, while TCGA-PDAC, GSE21501 and ICGC-PACA-AU were used for external validation. Subsequently, a nomogram integrating risk scores and clinical parameters was established, and gene set enrichment analysis (GSEA), tumor immunity analysis and drug sensitivity analysis were conducted.

**Results:**

A UPR-related signature comprising twelve genes was constructed and divided PDAC patients into high- and low-risk groups based on the median risk score. The UPR-related signature accurately predicted the prognosis and acted as an independent prognostic factor of PDAC patients, and the AUCs of the UPR-related signature in predicting PDAC prognosis at 1, 2 and 3 years were all more than 0.7 in the training and validation datasets. The UPR-related signature showed excellent performance in outcome prediction even in different clinicopathological subgroups, including the female (p<0.0001), male (p<0.0001), grade 1/2 (p<0.0001), grade 3 (p=0.028), N0 (p=0.043), N1 (p<0.001), and R0 (p<0.0001) groups. Furthermore, multiple immune-related pathways were enriched in the low-risk group, and risk scores in the low-risk group were also associated with significantly higher levels of tumor-infiltrating lymphocytes (TILs). In addition, DepMap drug sensitivity analysis and our validation experiment showed that PDAC cell lines with high UPR-related risk scores or UPR activation are more sensitive to floxuridine, which is used as an antineoplastic agent.

**Conclusion:**

Herein, we identified a novel UPR-related prognostic signature that showed high value in predicting survival in patients with PDAC. Targeting these UPR-related genes might be an alternative for PDAC therapy. Further experimental studies are required to reveal how these genes mediate ER stress and PDAC progression.

## Introduction

Pancreatic ductal adenocarcinoma (PDAC), accounting for over 90% of pancreatic cancer cases, is one of the leading causes of cancer-related death worldwide (1, 2). The incidence and mortality rates of pancreatic cancer have been on the rise for decades, with an estimated 62,210 new cases and 49,830 deaths in the United States in 2022 (3). Despite significant advances in conventional therapies and immunotherapy, the response rate and overall survival rate of pancreatic cancer are unsatisfactory (4). Less than 20% of patients are eligible for surgical resection, since the majority of pancreatic cancer cases are diagnosed at an advanced and unresectable stage with limited therapeutic options. Even after R0 resection, the 5-year survival rate is only approximately 15-25% (5, 6). Therefore, it is imperative to understand the underlying molecular mechanisms and develop more effective treatment strategies against it.

Cancer cells endure oncogenic and environmental stress, e.g., hypoxia and lack of nutrition, lactic acidosis, and oxidative stress that disrupts the endoplasmic reticulum (ER) and ultimately leads to the accumulation of unfolded or misfolded proteins in the ER, resulting in ER stress. Upon induction of ER stress, tumor cells evolve the unfolded protein response (UPR) as an intrinsic adaptive and prosurvival mechanism to restore ER homeostasis and overcome the hostile environment (7). In particular, the strategy of targeting the UPR is also highlighted to overcome the development of cancer (8). The UPR signalling pathway activates three ER stress sensors (ATF6, IRE1α, PERK) to attenuate translation, upregulate ER molecular chaperones, and induce ER-associated protein degradation, thereby mitigating ER workload, increasing ER folding activity, and promoting clearance of unfolded and misfolded proteins, respectively (9). Indeed, emerging evidence suggests that the activation of UPR has implicated many aspects of cancer cell biology, including invasion, angiogenesis, mitochondrial function, and tumor-associated inflammation, which contributes to the development of many cancers, including breast and prostate cancers (8). However, the role of the UPR pathway in the context of PDAC and its prognosis remains unclear and may represent an interesting avenue for thorough investigation.

In this study, we constructed a robust UPR-related signature and validated its function in independent cohorts of PDAC patients. We demonstrate that this novel signature can predict the prognosis of PDAC and is closely correlated with infiltrating immune cells and key immune checkpoints.

## Method

### Datasets and preprocessing

**Figure 1:**
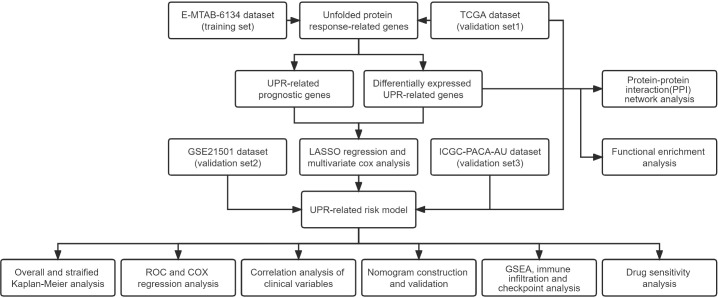
Flowchart of the study design.

A flowchart of this research is illustrated in **Figure 1**. The E-MTAB-6134 cohort was downloaded from the ArrayExpress database (https://www.ebi.ac.uk/arrayexpress/) and set as the training dataset. For the transcriptional profiles in the validation cohort, the RNA-seq and clinical data of 178 pancreatic cancer samples and 165 normal samples from The Cancer Genome Atlas (TCGA) and Genotype-Tissue Expression (GTEx) were retrieved from the UCSC Xena database (https://xenabrowser.net/datapages/). The GSE21501 dataset was obtained from the GEO (http://www.ncbi.nlm.nih.gov/geo). The clinical data of the PACA-AU cohort were collected from the ICGC data portal (https://dcc.icgc.org/). The clinical characteristics of the patients in the above cohort are outlined in **Supplementary Table 1**.

### Identification of the differentially expressed UPR-related genes

Using the R package “DESeq2”, the obtained raw read counts in TCGA were normalized, and 113 UPR-related genes obtained from the Molecular Signature database (https://www.gsea-msigdb.org/gsea/msigdb/) were extracted to identify the differentially expressed UPR-related genes with a false discovery rate (FDR) of<0.05. Protein–protein interaction (PPI) analysis was performed using the online network tool STRING (https://cn.string-db.org/). Cytoscape software (version 3.8.0) was used to build and visualize the PPI network.

### Functional enrichment analysis

The Gene Ontology (GO, http://www.geneontology.org) and the Kyoto Encyclopedia of Genes and Genomes (KEGG, http://www.kegg.jp/) were used to investigate potentially enriched functions and pathways of differentially expressed UPR-related genes *via* the “cluster Profiler” and “GOplot” R packages.

### Construction of a prognostic UPR-related gene signature

In this study, univariate Cox regression analysis was used to select genes with P<0.05 for subsequent dimension reduction analysis. Then, the least absolute shrinkage and selection operator (LASSO) regression algorithm was used to construct a signature by the R package “glmnet”. The regression coefficients (β) corresponding to the optimal λ value were incorporated into the prognostic signature formula as follows:

Risk score =(coefficient βi × expression of signature gene i)

Coefficient βi is the coefficient of gene i generated from LASSO regression analysis, and the expression of signature gene i is the transcripts per million reads (TPM) value of each selected gene. The risk score was calculated for each patient according to the formula, and the median was used to divide patients into high- and low-risk groups. Multivariate Cox analysis was further used to determine whether these genes are independent prognostic factors.

### Evaluation and validation of the accuracy of the prognostic UPR-related gene signature

Kaplan‒Meier survival curves were calculated between the two risk groups and was compared using the log-rank test. The receiver operating characteristic (ROC) and area under the curve (AUC) values corresponding to survival at 1, 2, and 3 years were used to assess the reliability and predictive ability of the prognostic signature. We performed univariate and multivariate Cox regression analyses of the risk score and other clinical variables to determine whether the risk score had independent prognostic value in PDAC patients. Correlation analysis was conducted to assess the relationship between the risk score and the clinical pathological characteristics of patients. Furthermore, we performed a stratified analysis to assess the precision of prognosis prediction based on other clinicopathological features.

### Construction and evaluation of a nomogram

A nomogram including the clinical parameters and risk scores in the E-MTAB-6134 dataset was constructed using the R package “rms”. Then, calibration curves were applied to compare the nomogram-predicted and actual OS probabilities *via* the R package “regplot”. By analyzing the DCA curve, the prediction performance of the nomogram, risk score, and other clinical parameters was assessed. Finally, the reliability of the nomogram was validated by TCGA dataset.

### Gene set enrichment analysis

Gene set enrichment analysis (GSEA) was used to explore potential molecular mechanisms associated with the risk score between the high- and low-risk groups by the R package “cluster Profiler”.

### Analysis of immune cell infiltration and immune checkpoint genes

Levels of 16 types of infiltrating immune cells and activity of 13 immune-related pathways or functions were determined using the R package “GSVA” through single-sample gene set enrichment analysis (ssGSEA). CIBERSORT was used to calculate the infiltration of immune cells in the samples and study the relationship between risk groups and immune checkpoints. Pearson’s test was used to evaluate the relationship between the signature risk score and the expression of immune checkpoint genes as well as DNA mismatch repair genes.

### Drug sensitivity analysis

All pancreatic cancer cell lines with expression data were downloaded from the Cancer Cell Line Encyclopedia database (CCLE, https://portals.broadinstitute.org/ccle) and classified into high- and low-risk groups based on the risk model. Then, the DepMap-PRISM Repurposing Secondary Screen database(https://depmap.org/portal/) was integrally utilized to identify cell line vulnerability to drugs.

### Cell culture

The pancreatic cancer cell lines AsPC-1 and MIAPaCa-2 were purchased from the American Type Culture Collection (ATCC). The AsPC-1 cell line was cultured in RPMI-1640 medium, and MIAPaCa-2 cells were cultured in DMEM. All medium was supplemented with 10% fetal bovine serum and 1% penicillin/streptomycin. In the case of MIAPaCa-2, the medium was supplemented with 2.5% horse serum (HS). DMEM, RPMI-1640, fetal bovine serum, horse serum, and penicillin/streptomycin were purchased from Gibco (Invitrogen-Gibco). Cells were maintained at 37°C with 5% CO^2^ in a humidified incubator.

### Plasmids and transfections

Human PDIA6 (NM-001282704), ZBTB17 (NM-003443), and ATF3 (NM-001674) overexpression plasmids were synthesized by HanYi Biosciences Inc. (Guangzhou, China). The SLC1A4 siRNA and negative control siRNA (Si-NC) were synthesized by JiMA Biotechnological, Inc. (Shanghai, China). The siRNA sequences were as follows: si-SLC1A4-1: 5’-GAGAAGAGCAACGAGACCATT-3’; si-SLC1A4-2: 5’-GUUGCAGCUUUCCGUACGUTT-3’. Transfections were performed using JetPRIME (Polyplus-transfection, France) according to the manufacturer’s instructions.

### RNA extraction and qRT-PCR

Total RNA extraction from AsPC-1 and MIAPaCa-2 cells was performed using TRIzol reagent (Yishan Biotech) and converted to cDNA by using Evo M-MLV RT Premix for qPCR (Accurate Biotechnology, Hunan, China). qRT-PCR analysis was conducted using the SYBR^®^ Green Premix Pro Taq HS qPCR Kit (Accurate Biotechnology, Hunan, China) with a Light Cycler 480 (Roche). The relative expression levels of target mRNAs were normalized to those of β-actin (ACTB), and expression fold changes were analyzed by the 2^-ΔΔCt^ method. Primers were synthesized by Sangon Biotech (Sangon, Shanghai, China). The sequences of the primers were as follows: PDIA6 forward, 5’-CCGCTGCAAGGTTAGGTCTC-3’; PDIA6 reverse, 5’-CGCCATCTACGCCTCACAAA-3’; ZBTB17 forward, 5’-TGTCTGGAAATCTGAGCCAT-3’; ZBTB17 reverse, 5’-CAGCTGCCGCTGCTGGTT-3’; ATF3 forward, 5’-ATCTCCTTCACCGTGGCTAC-3’; ATF3 reverse, 5’-AGGACCTGCCATCATACTGC-3’; SLC1A4 forward, 5’-ATTATGTGCTCAGCGACCCTTC-3’; SLC1A4 reverse, 5’-AACCTGCTGATCCTCTTGTCC-3’; ACTB forward, 5’-AGAGCTACGAGCTGCCTGAC-3’; ACTB reverse, 5’-AGCACTGTGTTGGCGTACAG-3’.

### Cell proliferation assays

Cell Counting Kit-8 (CCK-8, Dojindo, Japan) was used to assess cell viability as recommended by the manufacturer. Briefly, 5000 cells/well of AsPC-1 and MIAPaCa-2 cells transfected with siRNA (si-NC or si-SLC1A4) and PDIA6, ZBTB17, and ATF3 overexpression vectors or empty vector (Control) were cultivated in 96-well plates with five replicates and treated with floxuridine or dimethyl sulfoxide (DMSO) vehicle at the indicated concentration for 48 h. Then, CCK-8 reagent was added to the wells under light protection and incubated for 1.5 hours at 37°C and 5% CO2, and the absorbance at 450 nm was detected by microplate reader (Multiskan, Thermo Fisher Scientific, Inc.)

### Statistical analysis

All statistical analyses were carried out using the R (http://www.r-project.org) and R Bioconductor packages. A P value<0.05 was considered to indicate statistical significance.

## Result

### Identification and functional enrichment analysis of differentially expressed UPR-related genes in PDAC

**Figure 2:**
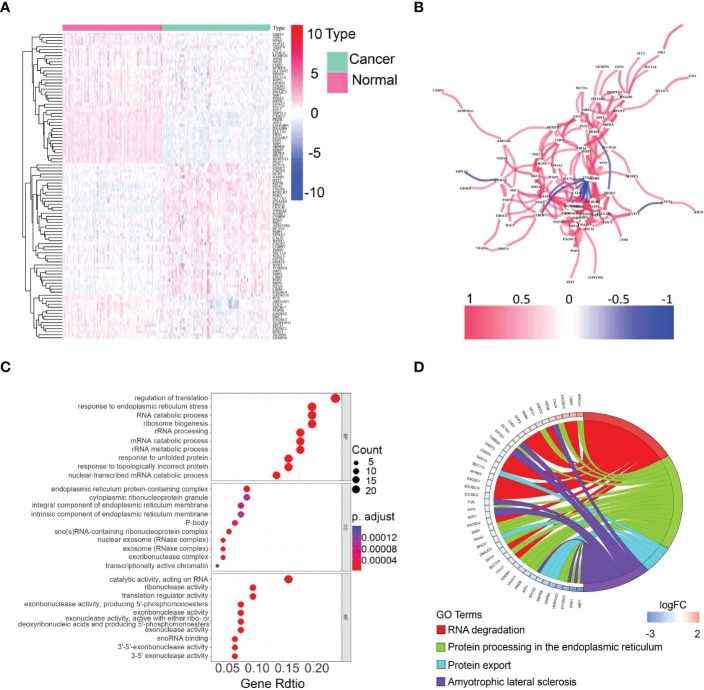
Identification of differentially expressed UPR-related genes and functional annotation. **(A)** A heatmap showing the expression of differentially expressed UPR-related genes in normal and cancer tissues in the TCGA. **(B)** The correlation network of selected candidate genes. **(C)** GO enrichment analysis. **(D)** KEGG pathway enrichment analysis.

The expression of 113 UPR-related genes was explored in The Cancer Genome Atlas (TCGA) data. In total, 101 differentially expressed UPR-related genes were identified between normal and cancer tissues, 43 of which were upregulated and 58 of which were downregulated (**Figure 2A**, **Supplementary Table 2**). Protein–protein interaction (PPI) network analysis and Pearson correlation analysis were used to explore the potential interactions among the differentially expressed UPR-related genes (**Figure 2B**, **Supplementary Figure 1**). The GO analysis results showed that the differentially expressed UPR-related genes were mainly enriched in regulation of translation, endoplasmic reticulum protein-containing complex, and catalytic activity acting on RNA (**Figure 2C**). The KEGG pathway enrichment analysis indicated that these genes had a close correlation with RNA degradation and protein processing in the endoplasmic reticulum (**Figure 2D**).

### Establishment of the prognostic risk signature in the E-MTAB-6134 training set

**Figure 3:**
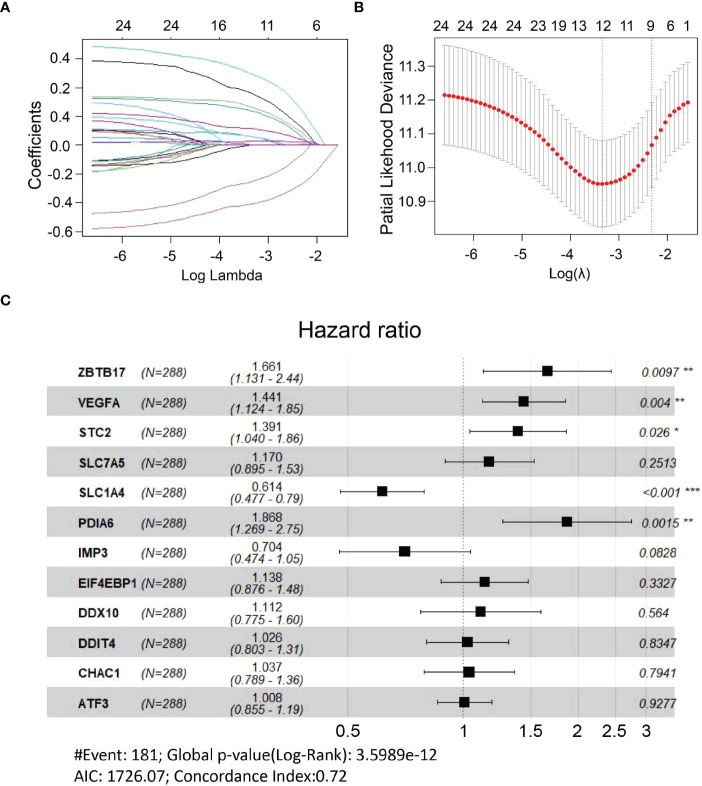
Construction of a UPR-related signature. **(A, B)** Candidate genes were screened using LASSO regression analysis. **(C)** Multivariate Cox regression analysis of candidate genes. *P<0.05, **P<0.01 and ***P<0.001.

All UPR-related genes in the E-MTAB-6134 dataset were included in univariate Cox regression analysis to identify 28 UPR-related prognostic genes (**Supplementary Table 3**). We further overlapped these genes with 101 differentially expressed UPR-related genes obtained from the TCGA dataset to identify 24 overlapping genes and retained them for further analysis (**Supplementary Figure 2**). LASSO analysis was performed to identify twelve UPR-related signature genes (ZBTB17, VEGFA, STC2, SLC7A5, SLC1A4, PDIA6, IMP3, ELF4EBP1, DDX10 DDIT4, CHAC1, ATF3) and generate risk scores according to the formula below (**Figures 3A, B**). The risk score = (0.507366×expression of ZBTB17) + (0.365323×expression of VEGFA) + (0.329991×expression of STC2) + (0.157084×expression of SLC7A5) + (-0.48772×expression of SLC1A4) +(0.624609×expression of PDIA6) + (-0.35054×expression of IMP3) + (0.129007×expression of EIF4EBP1) + (0.106289×expression of DDX10) + (0.026115×expression of DDIT4) + (0.036352×expression of CHAC1) + (0.007603×expression of ATF3). Next, we used multivariate Cox regression analysis to explore whether these genes are independent prognostic factors (**Figure 3C**).

### Validation of the UPR-related prognostic signature

**Figure 4:**
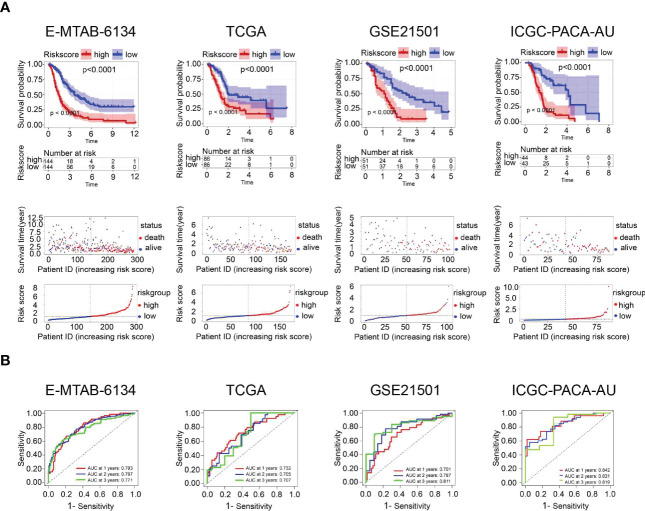
Prognostic performance of the UPR-related signature in the training dataset (E-MTAB-6134) and three validation datasets (TCGA, GSE21501, ICGC-PACA-AU). **(A)** Kaplan‒Meier survival curves, risk score distribution and survival status of patients in the high- and low-risk groups. **(B)** ROC curves were used to assess the predictive performance of the 1-, 2-, and 3-year OS.

All patients in the training dataset and validation datasets were classified into high- and low-risk groups according to the median risk score. Kaplan‒Meier curves indicated that the overall survival of the high-risk group was worse than that of the low-risk group. Furthermore, risk score distribution and survival status showed an increase in the death rate and a reduction in survival time along with the increment of risk scores, which was further confirmed by the validation datasets (**Figure 4A**). We also found that there was a significant difference in the expression of the twelve UPR-related genes between the high- and low-risk groups (**Supplementary Figure 3**). The ROC results demonstrated that the model has good accuracy in predicting 1-, 2-, and 3-year patient survival since all ROC curves had AUC values above 0.7 (**Figure 4B**).

### The clinical value of the UPR-related prognostic signature

**Figure 5:**
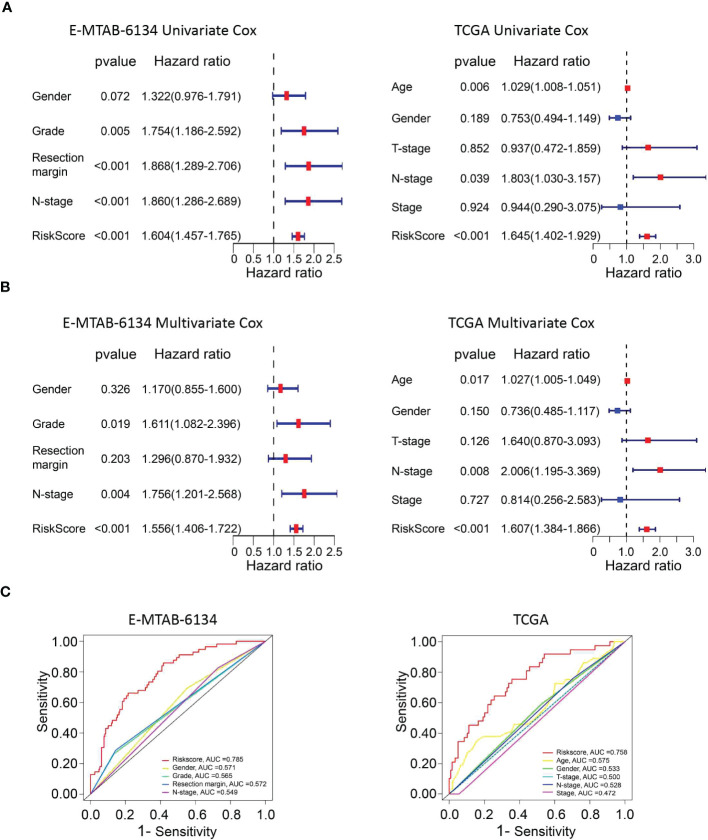
The predictive performance of the risk score and other clinicopathological parameters. **(A, B)** Validation of the risk score as an independent prognostic factor using univariate Cox analysis and multivariate Cox analysis in E-MTAB-6134 cohort and TCGA cohort. **(C)** ROC curves indicate the difference in AUC between clinical pathological features.

Univariate and multivariate Cox proportional hazard models were performed to identify the prognostic variables. Univariate Cox analysis demonstrated that grade (p=0.005), resection margin (p<0.001), N stage (p<0.001) and risk score (p<0.001) were related to adverse outcomes in the E-MTAB-6134 dataset. However, only the risk score (p<0.001) could serve as an independent prognostic factor, which was validated in the TCGA dataset (**Figures 5A, B**). The heatmaps showed that the expression of twelve UPR-related genes was also associated with the clinicopathological features of PDAC (**Supplementary Figure 4**). The UPR-related risk score had higher AUC values and was thus superior to the conventional clinicopathological parameters (**Figure 5C**).

### Correlation analysis and stratified analysis

**Figure 6:**
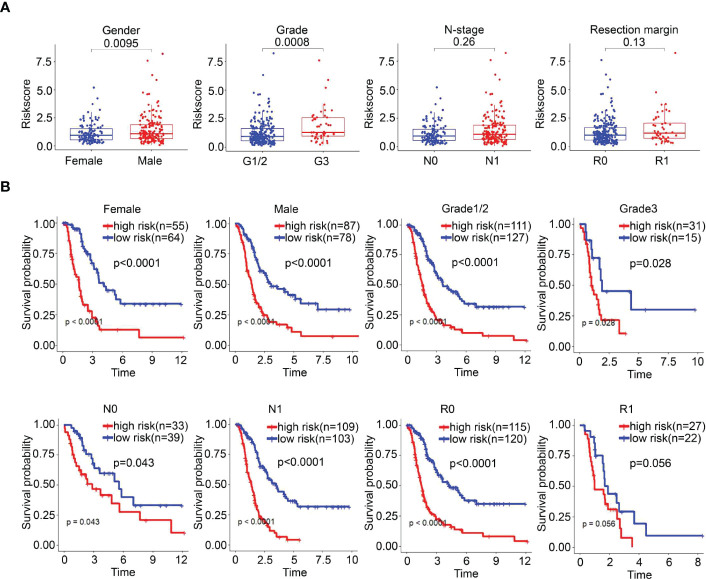
The survival outcomes of PDAC cancer patients in different risk groups. **(A)** Correlation between risk score and clinicopathological parameters. **(B)** Stratified analysis of the E-MTAB-6134 cohort. The survival outcomes of PDAC cancer patients with different risk scores in subgroups based on clinicopathological parameters.

The correlation between risk scores and clinicopathological parameters was investigated. The risk score was significantly related to sex (P=0.0095) and grade (P=0.0008), but it had no correlation with N stage or resection margin in the E-MTAB-6134 dataset (**Figure 6A**). Furthermore, we performed a clinical stratified analysis to study the survival outcomes of patients with different risk scores in different subgroups and found that the UPR-related signature showed excellent performance for survival outcome prediction in females (p<0.0001), males (p<0.0001), grade 1/2 (p<0.0001), grade 3 (p=0.028), N0 (p=0.043), N1 (p<0.001), and R0 (p<0.0001) (**Figure 6B**). The results above revealed that the UPR-related risk score signature was an effective predictor for survival in different clinicopathological subgroups.

### Construction and validation of a nomogram

**Figure 7:**
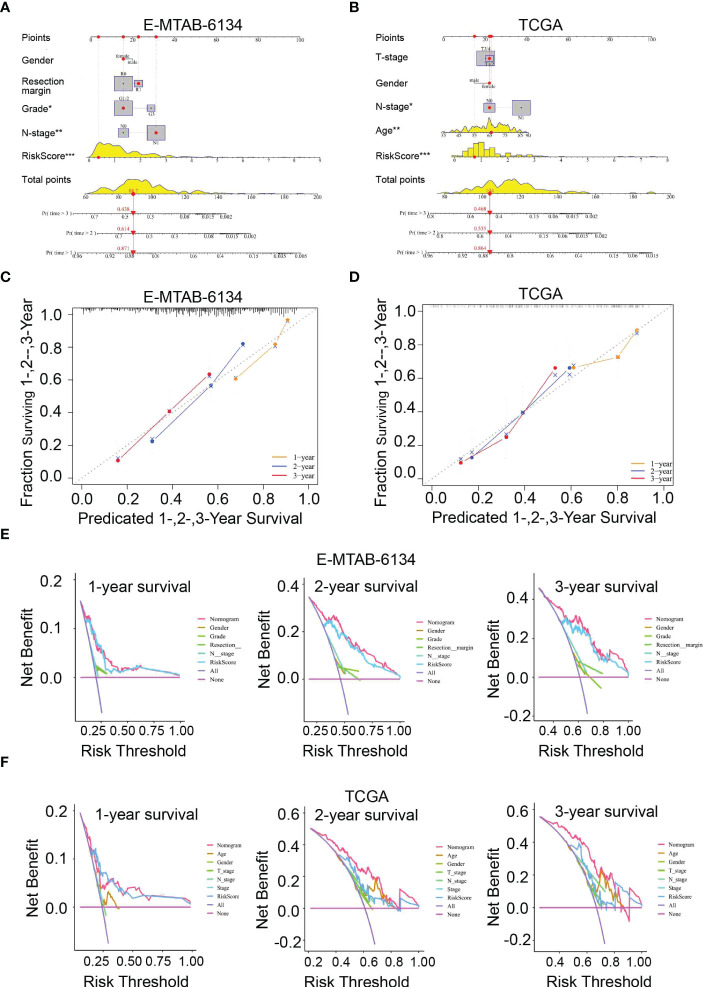
Nomogram for predicting 1-,2-, and 3-year OS. **(A, B)** Construction of a nomogram based on risk scores and other clinical factors. **(C, D)** The predictive accuracy of the nomogram verified by calibration curves. **(E, F)** DCA curves were used to compare the net survival benefit of the nomogram, risk score, and clinical parameters.

To better predict the survival outcomes of patients with PDAC, we constructed a comprehensive prognostic nomogram based on clinical data and risk scores. The nomogram showed that the risk score was the major factor affecting the prognosis of PDAC patients (**Figures 7A, B**). Moreover, the calibration curves of survival were highly consistent with the observations, while the DCA curves indicated that the nomogram was accurate and clinically reliable (**Figures 7C–F**).

### Functional analysis of the signature

**Figure 8:**
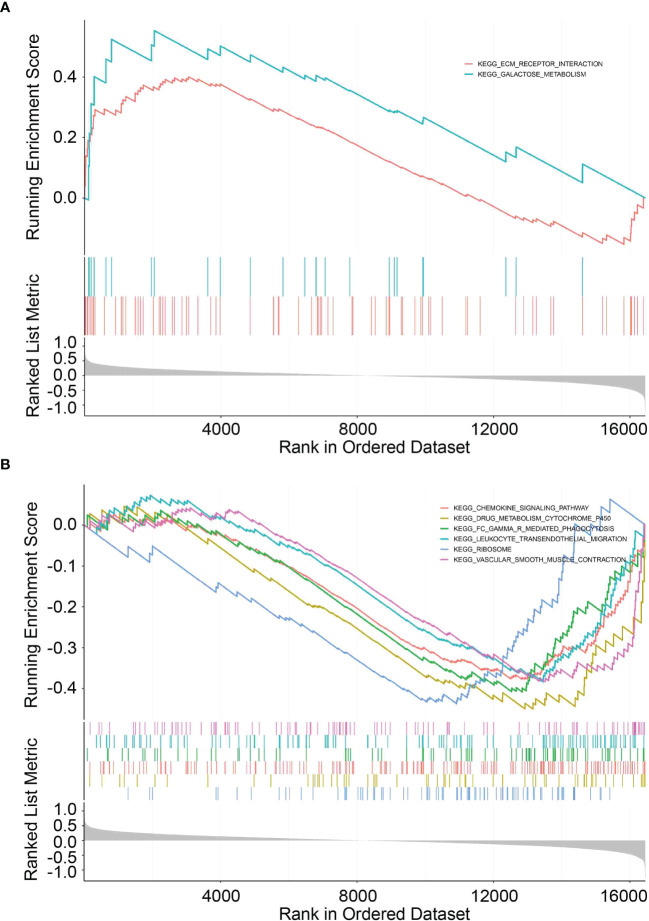
GSEA functional pathway analysis. **(A, B)** Top enriched biological pathways in high- and low-risk group patients.

To elucidate the potential function and pathways related to the prognostic signature, we performed GSEA to investigate the possible enriched signalling pathways between the two groups. The results showed that the high-risk group was mainly enriched in signalling pathways associated with ECM-receptor interaction and metabolism while the low-risk group was enriched in some immune-related pathways, indicating that the UPR signalling pathway is related to metabolism and immunity in pancreatic cancer (**Figures 8A, B**, **Supplementary Table 4**).

### Analysis of immune cell infiltration and immune checkpoint genes

**Figure 9:**
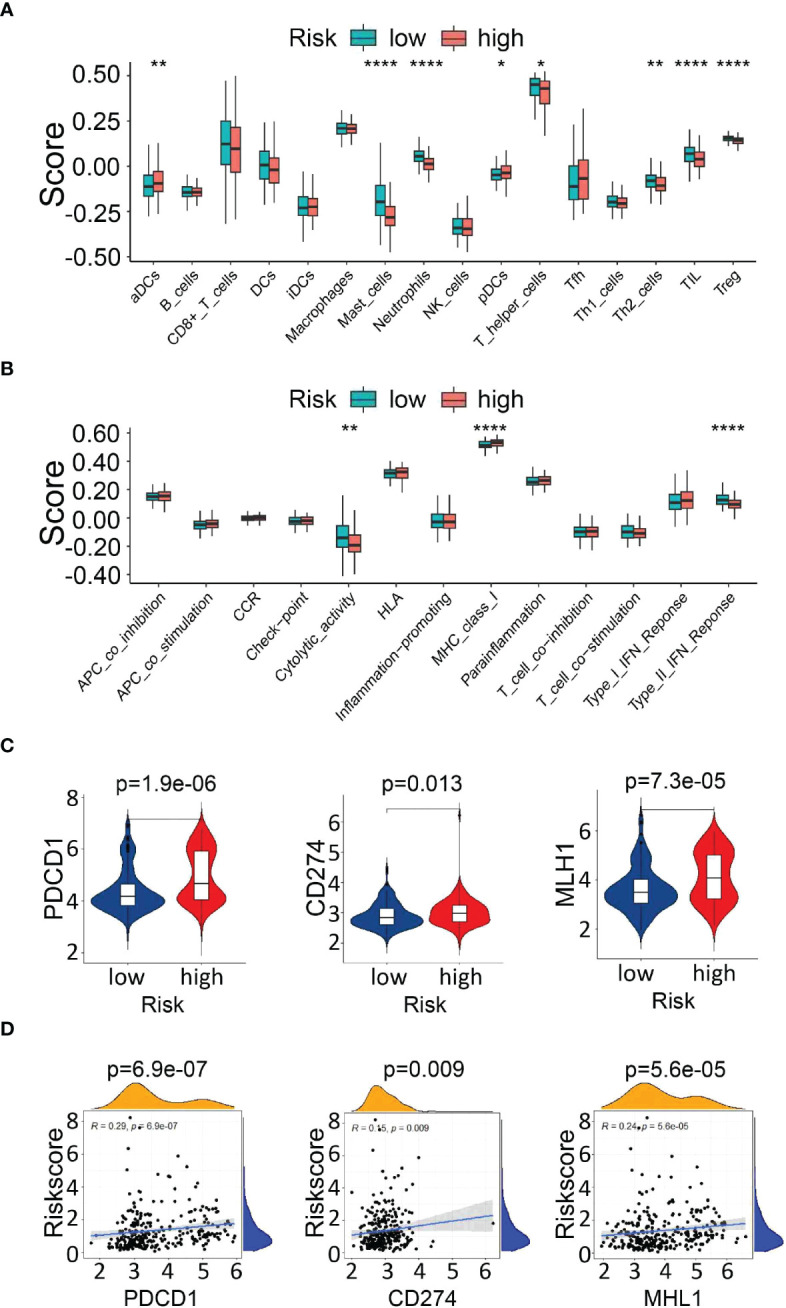
Analysis of immune cell infiltration and immune checkpoint genes. **(A, B)** Comparison of immune cells and immune-related pathways between the high- and low-risk groups. **(C)** Expression of immune checkpoint genes (PDCD1 and CD274) and the DNA mismatch repair gene MLH1 between the high- and low-risk groups. **(D)** The correlation between the risk score and the expression of PDCD1, CD274, and MLH1. *P<0.05, **P<0.01 and ***P<0.001.

To explore the correlation between the risk signature score and immune status, we performed enrichment analysis of immune cell types and immune-related pathways or functions by ssGSEA. Scores of mast cells, neutrophils, T helper cells, Th2 cells, tumor-infiltrating lymphocytes (TILs) and regulatory T cells (Tregs) were lower in the high-risk group than that in the low-risk group (**Figure 9A**). Furthermore, CIBERSORT was also implemented to assess the relationship of risk scores with 22 TIICs in PDAC, which verified that there was a significantly lower proportion of CD8+ T cells in the high-risk group (**Supplementary Figure 5**).

Next, the enrichment level of immune pathways related to cytolytic activity and type II FN response in the high-risk group was significantly lower than that in the low-risk group (**Figure 9B**). These results suggest that the decreased infiltration of tumor-infiltrating lymphocytes in the high-risk group might be partially due to reduced antigen delivery and recognition, and insufficient interferon gamma (IFN-γ) production.

In addition, we analyzed the relationship between the risk score and the expression of key immune checkpoints [PDCD1, CD274(PD-L1)] and DNA mismatch repair gene related to immunotherapy (MLH1), which showed elevated levels in the high-risk group, indicating that the unfavorable outcomes of high-risk patients might be due to the immunosuppressive microenvironment. (**Figures 9C, D**). The above findings indicated that the UPR-related signature model might play a potential role in predicting the immune response and tumor progression in PDAC patients.

### Validation for drug sensitivity

**Figure 10:**
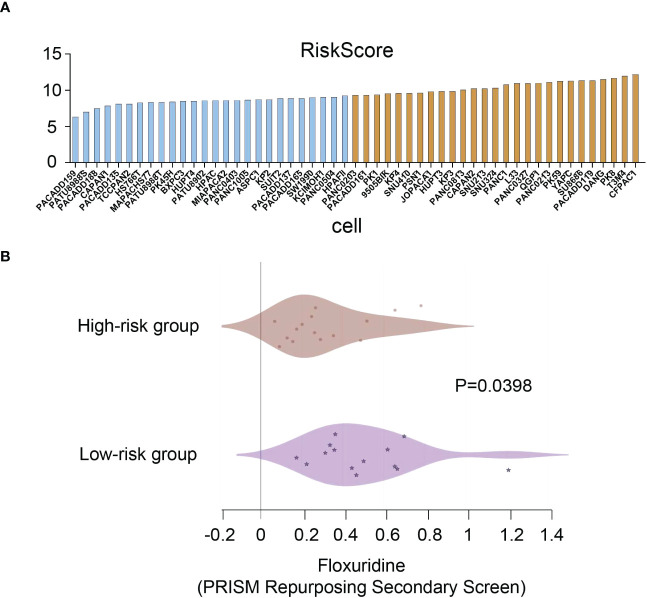
Drug sensitivity analysis. **(A)** Risk scores of pancreatic cancer cell lines. **(B)** Drug sensitivity values of floxuridine from the PRISM repurposing secondary screen of all pancreatic cancer cell lines.

**Figure 11:**
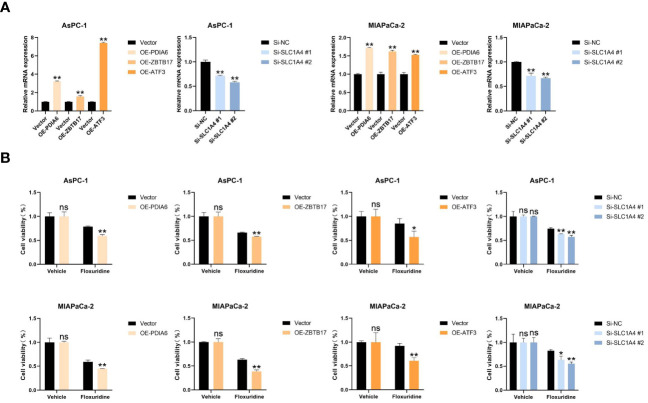
Cell proliferation assay. **(A)** Real-time RT‒PCR analysis of the mRNA levels of PDIA6, ZBTB17, ATF3, and SLC1A4 in the indicated cells. **(B)** Cell viability assay of the indicated cells treated with floxuridine. *P<0.05, **P<0.01. and ns=not significantly different (p > 0.05).

To identify potential drugs that might pave the way for the implementation of a targeted UPR-related signature for PDAC patients, we divided all pancreatic cancer cell lines with expression data into high- and low-risk groups according to our risk model (**Figure 10A**) and conducted drug sensitivity experiments. Drug sensitivity analysis with the DepMap-PRISM database showed that cell lines in the UPR high-risk group were more vulnerable to floxuridine than those in the low-risk group (**Figure 10B**). Subsequently, we conducted a CCK-8 cell viability assay to validate whether UPR-related genes were related to floxuridine sensitivity, and selected UPR-related genes, including PDIA6, ZBTB17, ATF3, and SLC1A4, to overexpress or knock down in AsPC-1 and MIAPaCa-2 cell lines, which are characterized as UPR-low-risk cell lines. Overexpression of PDIA6, ZBTB17, and ATF3 and knockdown of SLC1A4 were validated by qRT-PCR (**Figure 11A**). As shown in **Figure 11B**, overexpression of PDIA6, ZBTB17, and ATF3 and knockdown of SLC1A4 were more sensitive to floxuridine treatment than the vector control in both cell lines, which was in line with our analysis results.

## Discussion

Pancreatic ductal adenocarcinoma is a highly aggressive malignant tumor characterized by dismal survival in part due to limited surveillance methods and postoperative prognosis prediction, which highlights the need to identify reliable prognostic and predictive risk-stratification biomarkers (10). Currently, the prognosis evaluation of PDAC patients depends primarily on the TNM staging system; however, it remains inadequate for identifying the same stage discrepancy (11). Advances in the molecular biology of cancer have contributed to developing new predictive tools on the basis of prognosis-related genes. Considering the important role of the UPR, we used LASSO regression to identify a novel 12-gene UPR-related signature, which showed robust predictive performance for PDAC and better stratified the prognosis of patients with resected pancreatic cancer than the current generally applicable grading and staging methods (12). In addition, the integrated risk-assessment nomogram, which incorporates the UPR-related signature with other classical clinicopathological parameters, further improved the outcome prediction. To the best of our knowledge, the UPR-related prognostic signature and the nomogram described herein have not been reported previously. Importantly, our predictive model is based on the expression levels of selected genes, providing a smaller panel than whole-genome sequencing, which is more economical and clinically practical.

Previous studies have shown that the UPR pathway is required for cancer cells to maintain malignancy (7). Among them, ZBTB17 (MIZ1) could inhibit dsRNA uptake with MYC, thereby facilitating immune escape in PDAC (13). STC2 has been reported to be significantly stimulated under ER stress and to promote metastasis in pancreatic cancer (14, 15). DDIT4 has been discovered to trigger autophagy and aggravate ER stress (16). Furthermore, another study also verified that DDIT4 was related to poor prognosis in various tumors, including PDAC (17). Besides, VEGFA upregulation affects tumor progression caused by the induction of ER stress (18). In addition, a recent study in ATF3-deficient mice showed that loss of ATF3 prevents the initiation and progression of KRAS-mediated PDAC (19). Several studies have shown that elevated SLC7A5 expression predicts a poor prognosis in PDAC and is regarded as a potential biomarker (20–22). Consistently, our findings indicate that PDAC has a higher risk score and poorer prognosis, indicating the importance of genes associated with this signature. Additional studies are necessary to explore the precise roles of these identified genes.

To further elucidate the underlying mechanisms and related pathways by which the UPR-related signature mediates poor prognosis, we investigated the biological function of this signature. Based on Pearson correlation analysis, we observed significantly elevated MLH1 expression in the high-risk group. We thus speculated that the tumor cells of high-risk patients may easily escape the recognition of immune cells, and the infiltration of immune cells will be relatively poor. In addition, the higher expression of PD-1 and PD-L1 in the high-risk group indicated that there may be an immunosuppressive microenvironment. The biological processes above may ultimately contribute to a worse prognosis in the high-risk groups. As such, we hypothesize that the UPR may utilize immune mechanisms to promote the progression of pancreatic cancer. However, whether targeting the immune-related pathway could serve as an effective strategy for patients with high UPR-related risk scores still needs more in-depth exploration.

Despite great success in several cancer types, immunotherapy fails to elicit a response in PDAC due to its the unique tumor microenvironment (TME) (23, 24). The immunosuppressive microenvironment, which has features such as a lack of tumor-infiltrating lymphocytes and T-cell exhaustion, leads to the suppression of the anticancer immunity of PDAC (25, 26). In PDAC, CD8 T cells identify and kill cancer cells, indicating a favorable prognosis (27). Consistently, our GSEA results showed that the UPR signalling pathway is associated with immunity and that UPR high-risk patients were characterized by fewer tumor-infiltrating lymphocytes (TILs). Furthermore, an increased immune checkpoint was observed in the high-risk population, which suggests that the UPR-related signature could potentially be used to predict the immune microenvironment in PDAC.

In addition, our analysis and experimental results showed that cell lines with high UPR-related risk scores are more sensitive to floxuridine, a pyrimidine analogue that is used as a palliative treatment for patients with malignant neoplasms of the gastrointestinal tract. This finding indicated that patients classified into the high-risk group based on the UPR-related gene signature might potentially benefit from floxuridine treatment.

Nevertheless, the present study has certain limitations. First, model construction was driven by analysis of publicly available retrospective data with limited clinical information, and future studies with prospective validation are still needed. Second, the prognosis of PDAC patients is extremely poor, which leads to inaccurate results in predicting the long-term outcome. Third, the potential molecular mechanisms of the UPR-related signature genes in pancreatic cancer warrant further experimental studies.

In conclusion, our results show that the UPR-related signature participates in the tumor development and the immune microenvironment and may be a reliable prognostic marker and therapeutic indicator for PDAC.

## Data availability statement

The original contributions presented in the study are included in the article/**Supplementary material**. Further inquiries can be directed to the corresponding authors.

## Ethics statement

The studies involving human participants were reviewed and approved by The Eighth Affiliated Hospital, Sun Yat-Sun University. Written informed consent for participation was not required for this study in accordance with the national legislation and the institutional requirements.

## Author contributions

SL, LF designed and guided the study. SC, HG, SX carried out experiments, analyzed the data and wrote the manuscript. YL CZ, YC, JD contributed to processing pictures, interpretation of results. All authors contributed to the article and approved the submitted version.
