# Supplementary material for: Identification of an unfolded protein response-related signature for predicting the prognosis of pancreatic ductal adenocarcinoma
Source: Front Oncol. 2023 Jan 13;12:1060508. doi: 10.3389/fonc.2022.1060508 (PMC9885260; doi:10.3389/fonc.2022.1060508)
Supplement: Supplementary file 1 [file DataSheet_1.docx]

***Supplementary Material***

1. **Supplementary Figures**


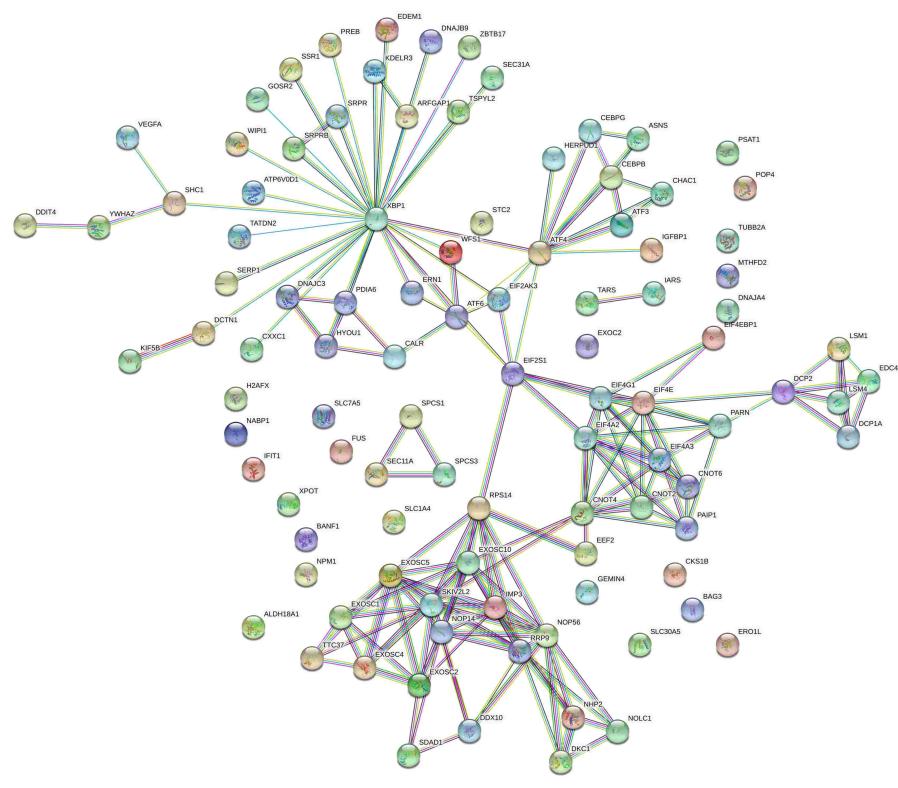


Figure 1 The interactions among candidate genes shown by the PPI network.


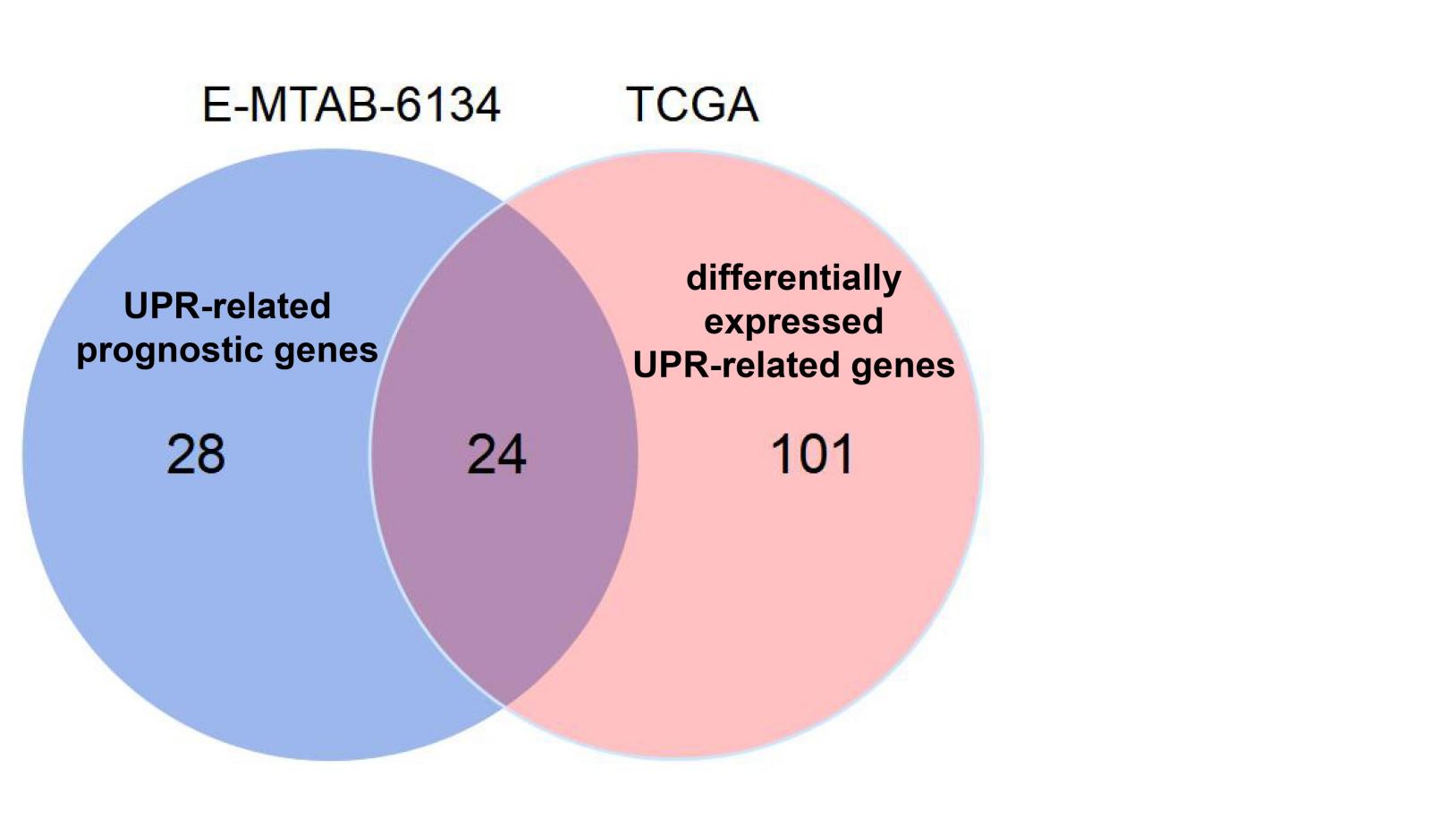


Figure 2 The UPR-related prognostic genes in the E-MTAB-6134 dataset overlapped with differentially expressed UPR-related genes in the TCGA dataset to obtain overlapping genes.

**
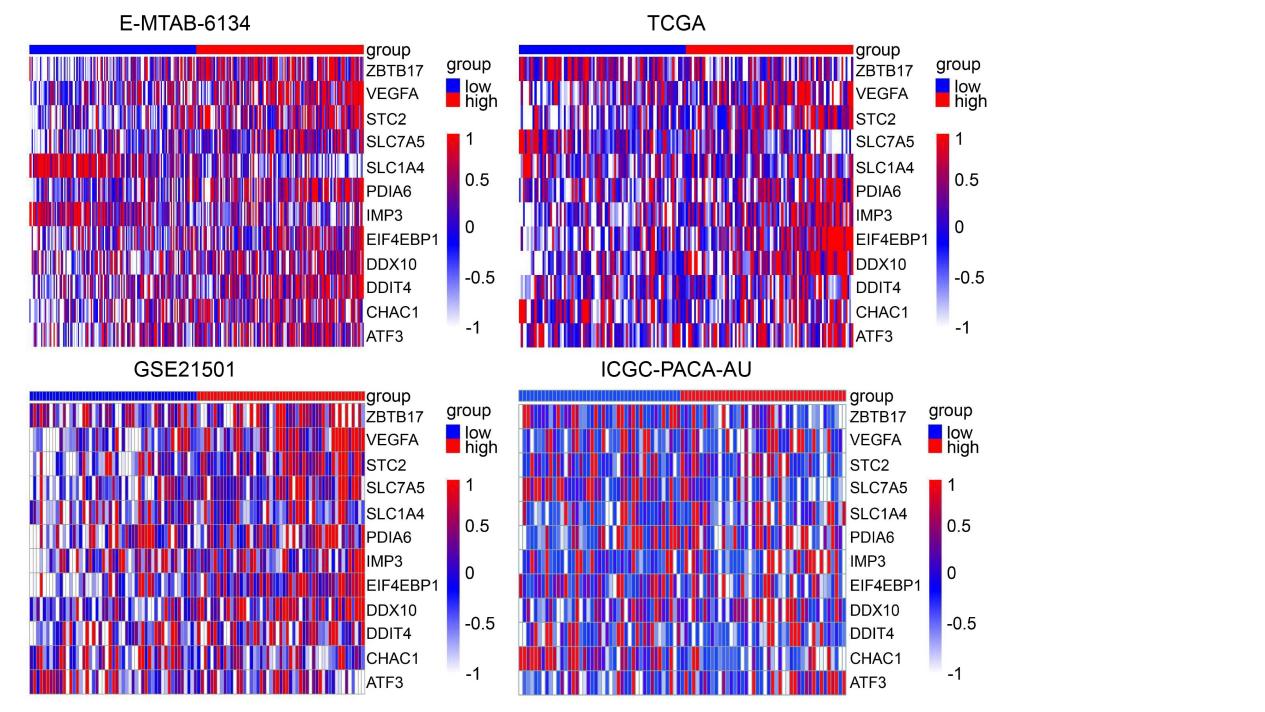
**

Figure 3 Gene expression heatmaps of the high- and low-risk groups.


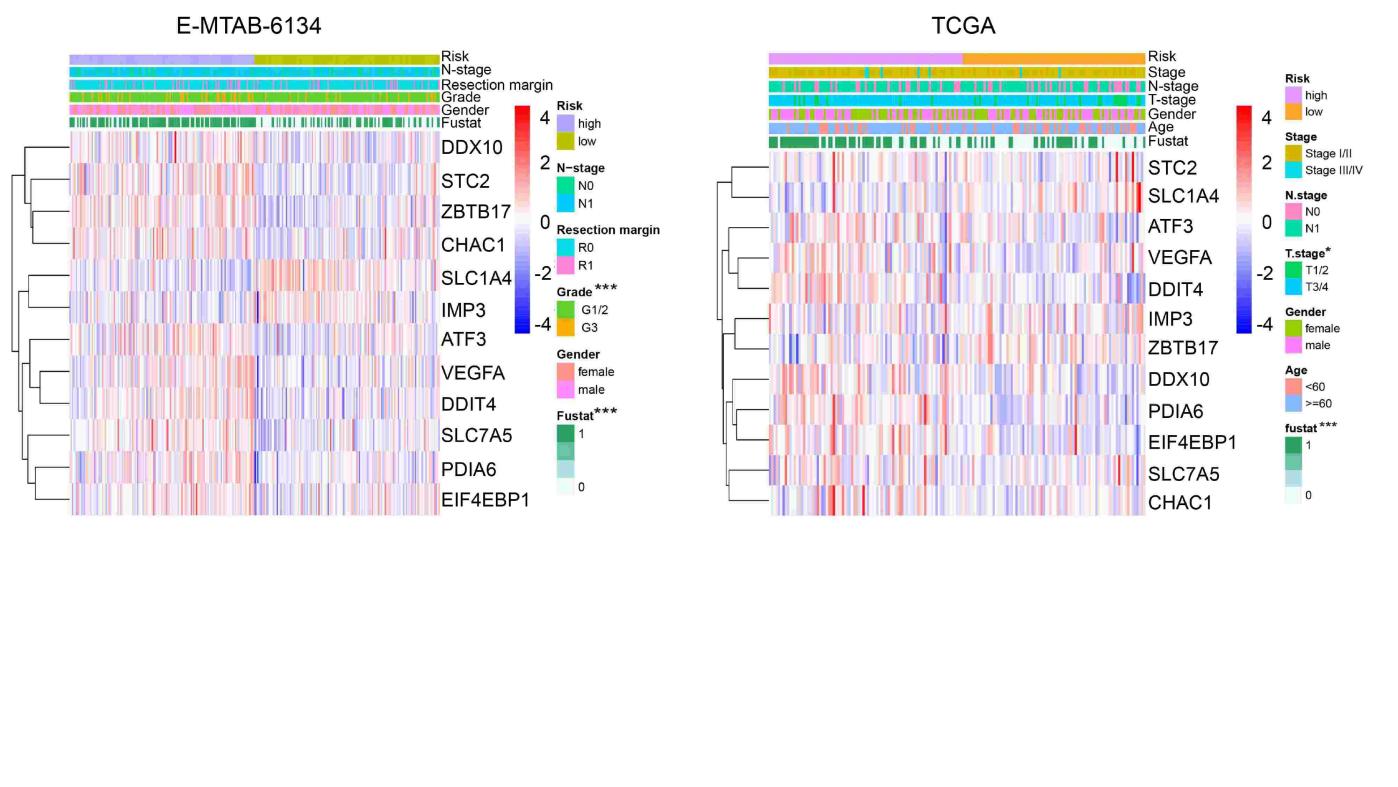


Figure 4 Multigroup heatmaps revealed the trends regarding signature gene expression and clinicopathological parameters.


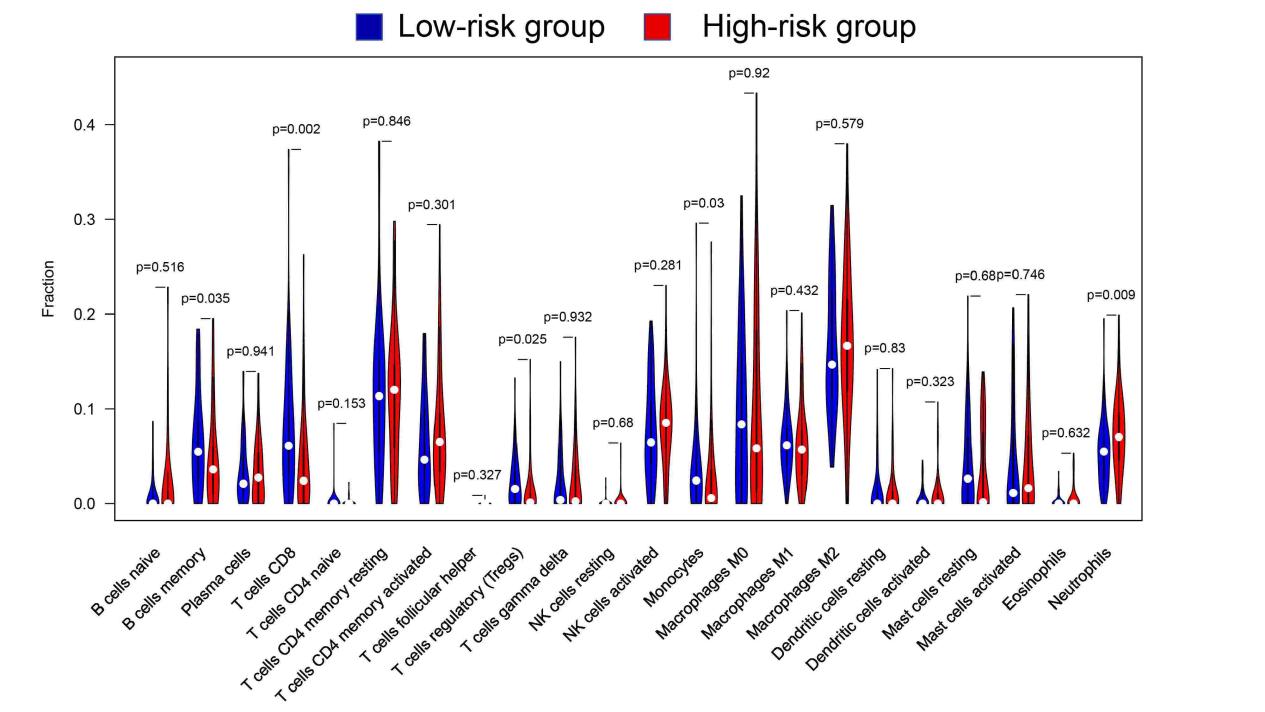
Figure 5 Violin plot indicating the difference in tumor-infiltrating immune cells between the high-risk group and low-risk group in the GSE21501 dataset.

1. **Supplementary Tables**

**Supplementary Table 1**

Clinical data of patients with PDAC in the training and validation datasets.

**Supplementary Table 2**

Differentially expressed UPR-related genes identified in the TCGA dataset.

**Supplementary Table 3**

Univariate Cox regression analysis of the UPR-related genes performed in the E-MTAB-6134 dataset.

**Supplementary Table 4**

Signalling pathways enriched in the high- and low-risk groups by GSEA.
